# Harnessing adaptive alleles in native animals for sustainable intensification of livestock

**DOI:** 10.1093/af/vfag024

**Published:** 2026-05-29

**Authors:** Muhammed Elayadeth-Meethal, Naseema Kolathingal-Thodika, Surinder S Chauhan

**Affiliations:** School of Agriculture, Food and Ecosystem Sciences, Faculty of Science, The University of Melbourne, Melbourne, VIC 3010, Australia; Kerala Veterinary and Animal Sciences University, Pookode, 673576, India; School of Agriculture, Food and Ecosystem Sciences, Faculty of Science, The University of Melbourne, Melbourne, VIC 3010, Australia; School of Agriculture, Food and Ecosystem Sciences, Faculty of Science, The University of Melbourne, Melbourne, VIC 3010, Australia

**Keywords:** adaptive genomics, haplotype introgression, hub-and-spoke, native livestock germplasm, sustainable livestock

## Abstract

**Graphical Abstract:**
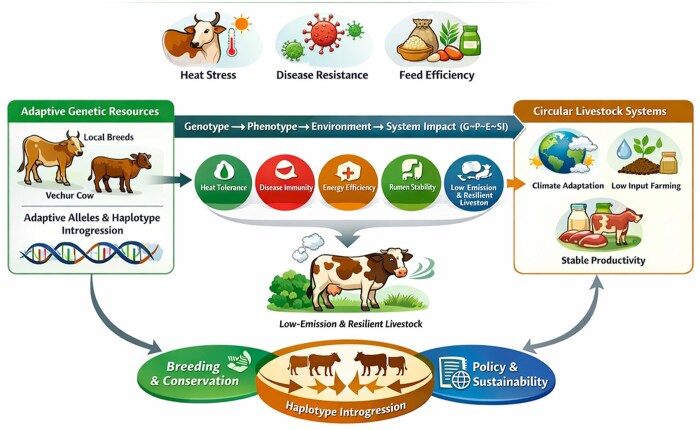

Implications

- Evidence from Global Farm Platform hub-and-spoke studies suggests that adaptable genetic variety can boost output while lowering reliance on external inputs in circular livestock systems.
- Native and locally adapted breeds are key genetic resources that could be used for climate adaptation and future food security.
- The genotype → phenotype → environment → system impact (G→P→E→SI) model predicts phenotypic resilience, environmental fitness, and stable system-level outcomes during climate stress.
- Directed haplotype introgression combines resistance and performance, resulting in minimal-input and low-emission livestock farming.

## Introduction

**Figure 1.**
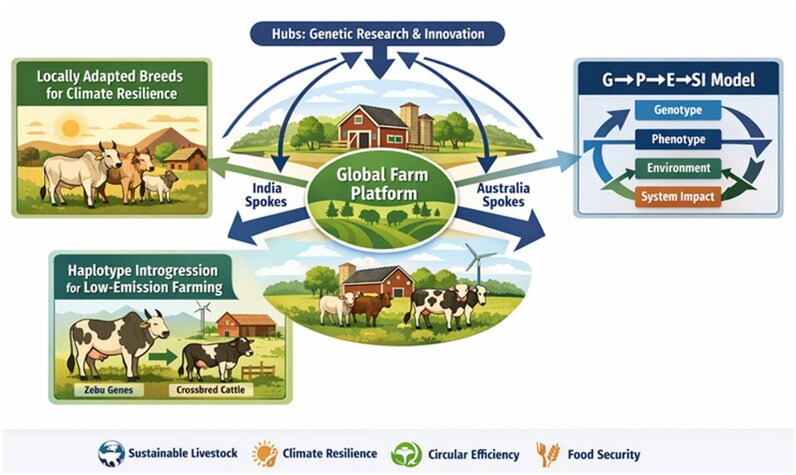
Adaptive genomics using a hub-and-spoke structure for circular and sustainable animal intensification. The diagram depicts how adaptive genetic asset found and defined at Global Farm Platform research hubs is converted into industrial and smallholder spokes to improve adaptability, circularity, and sustainability over time in livestock farming practices. Adaptive alleles, haplotypes, and selection signatures explain phenotypic features like heat tolerance, immunological capacity, energy efficiency, and rumen stability, all of which contribute to low-input production, a smaller environmental footprint, and climate stability across a variety of farming conditions.

Sustainable intensification strategies that provide productivity, resilience, and economic benefits while lowering dependency on external resources are becoming more important in global attempts to alter livestock systems under climate change (Eisler et al., 2014). In this regard, the hub-and-spoke Global Farm Platform (GFP) model offers a special global setting for combining agricultural practices, science, and policy across distinct agroecological zones (Figure 1) (Rivero et al., 2021, 2026; Lee et al., 2025). A vital but underutilized asset for sustainable livestock development, native and locally adapted cattle populations within these ecosystems in different climatic zones contain adaptive alleles, haplotypes, and selection signatures shaped by prolonged exposure to climatic pressure, feed restriction, and endemic disease (Elayadeth-Meethal et al., 2023; Kalaiyarasi and Elayadeth-Meethal, 2025). The present article, based on collaborative research conducted across GFP hubs-and-spokes in India, Australia, and the United Kingdom, presents evidence showing that adaptive variation in the genome underlying heat tolerance, immune competency, energy efficiency, and rumen stability leads to both phenotypic adaptation and system resilience. This synthesis shows how genomics-driven studies at GFP hubs can be applied through industrial and smaller-scale spokes to offer sustainable gains for producers and policymakers. This can be achieved by leveraging haplotype introgression (targeted breeding that combines the resilience of local breeds with the productivity of commercial lines) from the zebu and various locally adapted genotypes into commercial herds as a climate-adaptive production approach (Figure 1).

Different methods are being employed to achieve sustainable livestock intensification, with varying degrees of success. Several breeds have been recognized as potential candidates for enhancing resilience to impending global change caused by environmental perturbations. For example, Vechur, a renowned dwarf cow breed that originated in the southernmost region of the Indian subcontinent, is projected as a candidate breed in climate change scenarios (Eisler et al., 2014), as dwarf breeds possess functional traits for resilience (Elayadeth-Meethal et al., 2018). Also, the African Boran cattle are suggested for their *Theileria* tolerance (Wragg et al., 2022).

**Figure 2.**
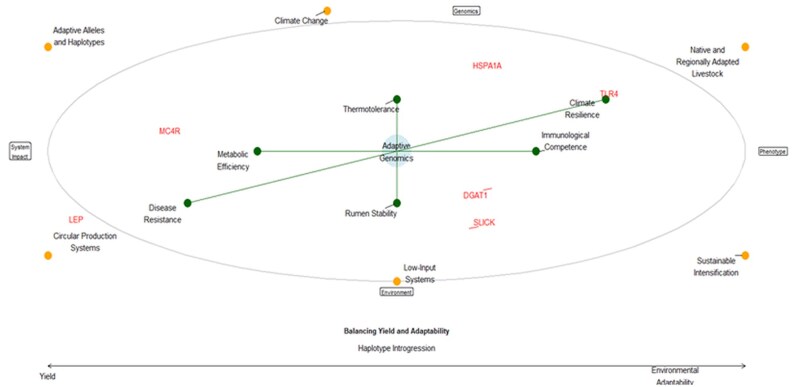
Adaptive genomics framework for sustainable livestock intensification integrating genetic diversity, environmental adaptation, and circular production systems. This framework demonstrates the use of adaptive genomics in sustainable cattle intensification. Key findings: 1) Adaptive alleles (such as HSPA1A, TLR4, and MC4R) improve climatic tolerance and efficiency. 2) The G → P → E → SI strategy connects genomic features to systemic sustainability and circularity. 3) Haplotype introgression balances yield and environmental adaptability in a variety of production systems.

Identifying adaptive livestock germplasm provides the foundation for research into the genetic architecture of the desired trait, as well as the genetic basis of trait development and how those qualities might be better integrated into livestock intensification for sustainability. A schematic framework illustrating the integration of adaptive genomics into a sustainable and circular production system is shown in Figure 1. The goal of this review is to synthesize existing evidence on adaptive genomic variation in livestock, with a focus on adaptive alleles, haplotypes, and selection signatures found in native and locally adapted cattle populations, and to assess their significance for sustainable livestock intensification under climate variability (Figure 2). Specifically, this synthesis seeks to 1) sum up present understanding regarding the evolutionary and genomic foundation for climate-resilient traits, like thermotolerance, energy efficiency, and disease adaptation; 2) bring together genomic, phenotypic, and environmental viewpoints using a genotype → phenotype → environment → system impact (G→P→E→SI) model; 3) examine the significance of haplotype variant hybridization and introgression as methods for transferring adaptive resistance to climate resilience.

## Evolution of Traits under Domestication Pressure

On an evolutionary scale, domestication pressure imposed by natural selection has led to the evolution of traits in cattle. A variety of morphological and cognitive alterations that define domestic cattle as distinct from their wild predecessors are the outcome of this phenomenon, which is referred to as “domestication syndrome” (Gleeson and Wilson, 2023). Significant physical and physiological changes have resulted from the domestication of cattle, which started about 10,000 yr ago. For example, compared to their wild ancestors, modern cattle have smaller brains, different hormone profiles, and more docility. Both deliberate human selection and unintentional selection brought on by the captive environment are likely to be responsible for these modifications (Elayadeth-Meethal et al., 2018). Many loci under selection in domesticated cattle have been found through genomic analysis, including genes related to ­immunology, metabolism, and reproduction (Wragg et al., 2022).

In response to the environmental conditions that existed throughout the domestication phase, animals were naturally selected for several traits, including an emphasis on disease resistance and climate resilience (Elayadeth-Meethal et al., 2018; Wragg et al., 2022). These important features have proven to be critical for domesticated animals’ survival and productivity in a range of agricultural situations (Wragg et al., 2022). Since domesticated animals are more likely to get exposed to pathogens due to increasing population density and human proximity, disease resistance has been a major focus of selection (Kalaiyarasi and Elayadeth-Meethal, 2025).

The ability to adapt to a changing climate is becoming more crucial in the face of global climate change. Heat tolerance and adaptation to multiple environments have been found in a variety of domesticated species, including tropical-adapted cow breeds (Eisler et al., 2014). Recent research has also shown that indigenous breeds can serve as repositories of genetic variation for climate adaptation features. The interaction of these traits and environmental factors throughout domestication established the genetic architecture of domesticated livestock (Elayadeth-Meethal et al., 2018). Advanced genomic tools, including whole-genome sequencing and genome-wide association studies, give insights into the genetic foundations of these adaptations, which were previously inaccessible (Eisler et al., 2014). Several measurement tools are also suggested for evaluating the efficacy of interventions (e.g., proxies for methane phenotypes, Kolathingal-Thodika et al., 2025). These can be integrated into the existing trials in GFP Hubs.

## Convergent Evolution of Traits in Different Environments

In the African subcontinent, the genetic diversity of cattle breeds has been investigated using several methodologies to determine the evolutionary origin of breeds and traits. The mitochondrial genome investigation of 90 African, European, and Indian breeds indicated transcontinental clustering and substantial divergence between the *Bos taurus* and *B. indicus* breeds. The genus *Bos* diverged around 11 million yr ago from a common ancestor with bison. Later divergence happened among *Bos indicus* and different ancestral lines at ∼117,000–275,000 yr before present (BP) and among European and African lineage groups at ∼22,000–26,000 BP (Bradley et al., 1996). Interestingly, domestication-associated trait development occurred later than the divergence of Indian, African, and European bovine lineages (Elayadeth-Meethal et al., 2018).

Expanding on this evolutionary framework, the genetic architecture of adaptation and production-associated traits has been widely studied, notably in cow breeds native to the Indian subcontinent. Several studies have investigated genomic areas, candidate genes, and biochemical markers linked to thermotolerance, energy efficiency, and fitness-associated traits in native and miniature cattle breeds (Dash et al., 2024). In dwarfs, multiple potential genes and molecular markers suggest an evolutionary genetic foundation for tolerance features such as heat stress adaptation and metabolic regulation (Elayadeth-Meethal et al., 2021, 2023; Elayadeth-Meethal and Kolathingal-Thodika, 2025). Gene markers associated with resilience to hemoprotozoan diseases and parasites, such as tolerance to trypanosomiasis and tick resistance, demonstrate native germplasm’s adaptive capacity (Kalaiyarasi and Elayadeth-Meethal, 2023).

Advanced genomic technologies have favored fine-tuning the finding of trait-associated genetic architectures. Recent research has found selection signatures for traits linked with sustainable intensification in Gir and Holstein cows, Red Sindhi, and Indian and Asian zebu cows (see Chishi et al., 2025). Integrating a global perspective enables the discovery of both shared and unique adaptable genetic traits, offering lessons for breeding methods that combine performance with environmental adaptability in a variety of climatic and farming environments. Global screening of adapted alleles revealed adaptive genetic fingerprints for several economically significant traits, including disease tolerance. For instance, a 6 Mb genomic region on chromosome 15 was found to be associated with *Theileria* tolerance in African Boran cattle (Wragg et al., 2022). Fas-associated factor 1 (FAF1), hemoglobin subunit beta, and the olfactory genes OR51G1 and OR51H1 were among the candidate genes identified in this location. Further OR51H1 gene screening of Vechur (*B*. *indicus*) and crossbred (*B*. *taurus* × *B*. *indicus*) cows revealed five single nucleotide variations (SNV) in Vechur cattle associated with tolerance to *Theileria* natural infection (Kalaiyarasi and Elayadeth-Meethal, 2025), demonstrating the convergent evolution of traits in globally distributed populations.

**Figure 3.**
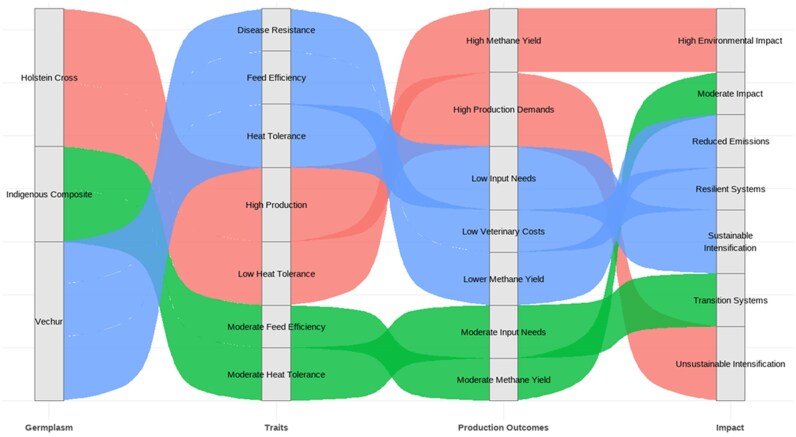
The integrated Sankey framework connects native germplasm, adapted alleles and haplotypes, functional features, and system-level results, demonstrating paths spanning adaptive genomics to sustainable livestock intensification. Genetic resources are transferred from native germplasm to sustainable intensification. Native and crossbred cattle have adaptive haplotypes that affect phenotypic and system resilience across climate zones. The zebu introgression increases heat resilience, disease resistance, and low-input efficiency, illustrating how genetic adaptation can allow sustainable livestock intensification under dynamic environmental circumstances.

Particularly, recognizing breed-level genetic variation lays the groundwork for determining the way these adaptive traits translate to system-level sustainability benefits (Figure 3). The review uses a genotype, phenotype, environment, and system impact (G→P→E→SI) approach, which connects adaptable alleles and haplotypes to functional features, environmental fitness, and overall production outputs. This methodology allows for the integration of genetic, phenotypic, and environmental data to demonstrate how native genotypes and haplotype introgression can help to create climate-resilient, minimal-input, and low-emission livestock systems (Lee et al., 2025). By including genetic architecture into this system’s viewpoint, the approach underlines not only the molecular foundation of adaptation but also its practical implications for long-term intensification strategies (Oke et al., 2025). To achieve these goals, the GFP Hub-and-Spoke network offers coordinated workflow (Rivero et al., 2026).

**Figure 4.**
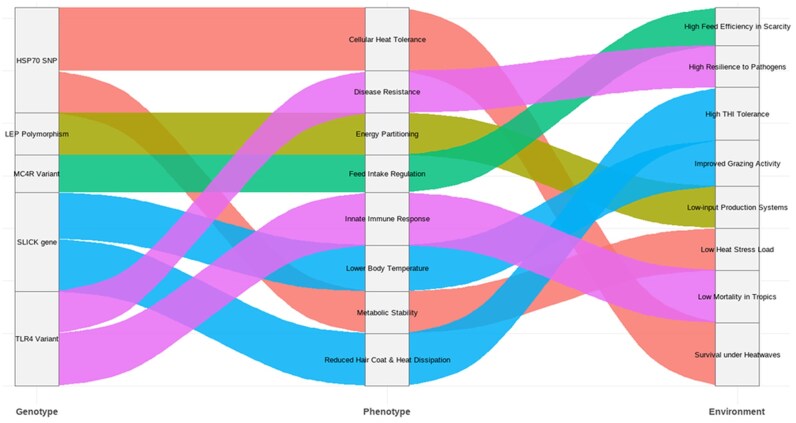
Genotype, phenotype, and environment mapping in adaptive cattle. The schematic shows how different genotypes contribute circularity and sustainability in different environments.

While much of the study on adapted alleles and haplotypes has been on native breeds from tropical and subtropical regions, adaptive genomics is equally applicable to temperate and crossbred livestock systems (Figure 4) (see Strillacci et al., 2025; Yadav et al., 2025). Studies from Europe, North America, and various temperate areas show that regionally adapted breeds and crossbred herds have genetic variation that determines heat stress resilience, metabolic efficiency, and disease tolerance across environmental conditions (Gao et al., 2025). Comparative assessments of indicine, taurine, and crossbred populations show that adaptive alleles can be selectively introduced to improve climate resilience and low-input efficiency in various circular systems of production (Goli et al., 2025). Utilizing these unidentified alleles and haplotypes can be facilitated by scaling up the existing GFP Hub-and-Spoke design (Lee et al., 2025).

Besides the conservation and use of native germplasm, crossbreeding and directed haplotype introgression provide feasible options for incorporating adaptive genetic diversity into larger production systems (Hamilton and Miller, 2016). Significant adaptation variants from locally evolved breeds can be introduced into temperate or high-producing herds to improve heat tolerance, immune competence, energy efficiency, and rumen stability while maintaining overall productivity (Elayadeth-Meethal et al., 2018). Such strategies allow for the creation of low-input, climate-tolerant herds that mix the strength of native breeds with the performance characteristics of commercial lineages, as in Indonesian cattle (Wang et al., 2025). Crossbreeding and introgression in the G → P → E → SI paradigm channel genetic potential from alleles, haplotypes, and selection signatures across functional features, resulting in beneficial environmental fitness consequences and system-wide stability. Presenting trait-directed introgression as a purposeful, genomics-based approach emphasizes its importance for breeding initiatives as well as for global policy choices about sustainable livestock intensification. However, significant constraints remain, including the scarcity of whole-genome data (Wang et al., 2025). A pangenome strategy using GFP Hubs-and-Spokes can be tested to accomplish an economically viable scale-up (Eisler et al., 2014).

Breeding strategies, genetic management of resources, and conservation goals are all significantly impacted by adaptive genomics in farming. Policymakers and researchers can create climate-smart methods that improve resilience, production, and environmental sustainability by finding and strategically employing adaptable alleles and haplotypes from local germplasm. While haplotype introgression and genomic-informed selection can maximize the spread of adaptation traits into commercial herds, conservation of regionally adapted breeds guarantees the long-term accessibility of vital genetic assets. Low-input production, circular climate-resilient livestock systems, and sustainable intensification are all supported by incorporating these strategies into regional and national programs. Strategies such as early-life programming can be stacked on these to improve the performance (Kolathingal-Thodika et al., 2025). This viewpoint emphasizes how crucial it is to connect genomic science with useful breeding and management resources, allowing for integrated strategies to achieve global food security and sustainability targets (Eisler et al., 2014).

## From Breeds to Traits: Exploring Genetic Architecture

**Figure 5.**
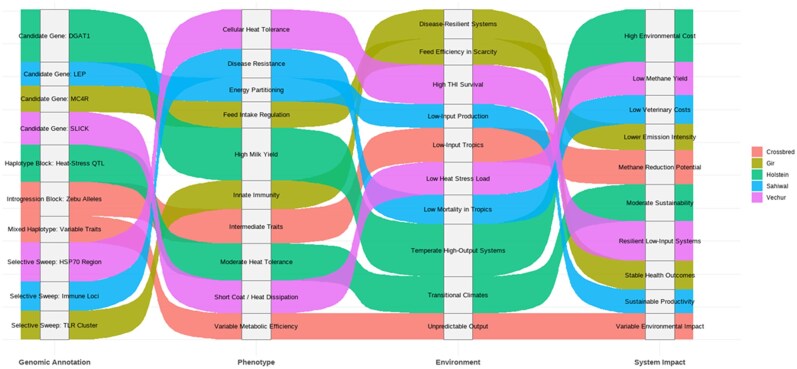
The causal and functional flow from adaptive genetic diversity to system-level impacts in cattle emphasizes the importance of native germplasm in supporting climate-resilient livestock systems. The G → P → E → SI route is shown schematically. Flows are colored by breed, with high-impact alleles emphasized. Zebu introgression into crossbred and temperate breeds is shown to demonstrate how adaptive genetic variants influence functional features, environmental fitness, and system-level results (e.g., low-input resilience, reduced methane, high productivity).

Although higher taxa and breed divergence is evolutionarily old, as demonstrated by modern omics approaches, trait evolution is comparably newer and occurs along with domestication. For instance, although divergence of *B*. *indicus* and *B*. *taurus* occurred 0.92 Myr ago, the ancestors of locally adapted breeds in South and North India originated at about 30,000–200,000 yr ago (Elayadeth-Meethal et al., 2018). Specifically, most of those traits originated in response to the respective environments and domestication needs (Figure 5). For Vechur cattle, the smaller body size was attributed to the parallel evolution of heat tolerance in a hot and humid environment prevailing in the home tract where the breed originated (Elayadeth-Meethal et al., 2018).

## Delineating the Genetic Architecture of Traits

Widespread use of omic techniques in animal breeding has enabled the identification of the evolutionary history and genetic architecture of adaptive and economic traits at the genetic and genomic levels. The mechanisms varied from the identification of alleles at specific loci (genetic polymorphisms like SNPs-single nucleotide polymorphisms), InDels-insertion-deletion to chromosome segments (genetic signature). Globally, several studies have been undertaken in different production systems in a similar line, providing global access to trait-associated alleles and candidate genes. Holstein Friesian cattle, selected primarily for milk yield, harbor genes supporting production efficiency; however, the genetic diversity is less in Holstein compared to locally adapted herds such as Gir (India) and dairy Gir (Brazil) (Braga et al., 2025). This low genetic diversity in Holsteins is reflected in the form of inbreeding depression with high runs of homozygosity (ROH), with longer ROH classes over 8 Mb, and rebounded in commercial herds as negative effects on yield (Ablondi et al., 2023). In Holsteins, the low genetic variability also led to metabolic diseases such as ketosis and lower resilience (Braga et al., 2025). The heritability is moderate to high for Holsteins for lifetime milk production and longevity in tropical climate which suggests room for genetic improvement (Saleh et al., 2026). Genomic regions harboring genes such as DGAT1, EXOSC4, PPP1R16A, and FOXH1 that regulate milk yield and composition traits have been identified in Holsteins and local cattle herds (Cortes-Hernandez et al., 2025). Polymorphisms in the leptin (LEP) gene were also associated with milk yield and composition traits (Yadav et al., 2023). Cows harboring SLICK gene variants are heat-tolerant, and SLICK gene-edited cows have been proposed as an alternative for selection for heat tolerance (Altman et al., 2025). Through GFP Hub-and-Spoke, Zebu and locally adapted genotype introgression into crossbred and temperate breeds is proposed, illustrating how adaptive genetic polymorphisms can introduce functional features, environmental fitness, and system-wide outcomes (e.g., low-input resilience, reduced methane output, and high productivity).

## Conclusions

Adaptive genomics provides a revolutionary approach for sustainable livestock intensification by relating alleles and haplotypes in native and crossbred herds to functional features, environmental fitness, and system-level outputs. Native genotypes contain a wealth of adaptive variation in genes that can improve heat tolerance, disease immunity, energy efficiency, and rumen stability, hence enabling low-input and climate-resilient production systems. The Global Farm Platform Hub-and-Spoke research architecture allows genomic insights developed at research hubs to be reviewed and adapted across varied industry and smallholder spokes, increasing their application throughout production settings in the global south or temperate zones. Integrating these findings into the Genotype → Phenotype → Environment → System Impact (G → P → E → SI) framework demonstrates how genetic adaptation leads to efficiency, ecological sustainability, and adaptability in varied systems of production. We present a compilation of studies on climate resistance features in livestock, most of which are the result of coordinated hub-and-spoke experiments across different agroecological zones. We also identify future collaborative research opportunities to explore trait-associated alleles and other genetic markers that may be useful to generate herds of cattle suitable for a changing climate in respective environments and production systems. Looking ahead, intentional haplotype introgression and hybridization programs offer an achievable means to combine the resilience of locally adapted varieties with the productivity of commercial herds. The hub-and-spoke model provides a means for continual evaluation, improvement, and implementation instead of rapid massive adoption. Genomic-based selection shapes breeding policy and conservation objectives. Future studies should focus on expanding global comparative assessments of adaptable alleles, improving systems-level models of trait–environment interactions. This will develop integrated decision-support systems for circular, climate-smart, low-input, and low-emission livestock production. Overall, adaptive genomics provides a conceptual and practical way to ensure sustainable, resilient, and productive livestock farms in an evolving setting, relying on global hub-and-spoke linkages for scaling.

*Conflict of interest statement*. The authors declare no real or perceived conflicts of interest.

## Author Biographies

Dr Muhammed Elayadeth-Meethal is a researcher in animal breeding, genetics, and biostatistics, currently based at the School of Agriculture, Food and Ecosystem Sciences, Faculty of Science, University of Melbourne. His research focuses on sustainable livestock intensification through the integration of genetics, genomic selection, data analytics, and environmental assessment. He has worked collaboratively with producers, researchers, and industry partners in India, Australia, and the United Kingdom. Previously, he served as Assistant Professor of Animal Breeding, Genetics and Biostatistics at Kerala Veterinary and Animal Sciences University, India. He was also awarded a Crawford Fund Fellowship at the University of Western Australia and a Rothamsted International Postdoctoral Fellowship at Rothamsted Research, United Kingdom. He holds a Bachelor of Veterinary Science and Animal Husbandry and master’s and a PhD in Animal Breeding, Genetics and Biostatistics. His current work explores stacking genomic selection with complementary interventions to select climate resilient livestock for sustainable production.

Naseema Kolathingal-Thodika is a PhD candidate in the School of Agriculture, Food and Ecosystem Sciences, Faculty of Science, University of Melbourne. Her doctoral research investigates innovative approaches, including foetal and early-life programming, to support climate-resilient livestock production and sustainable livestock intensification. She is the recipient of the prestigious Elevate Doctoral Fellowship from the Australian Academy of Technological Sciences and Engineering (ATSE). She also received a postgraduate student award from the Crawford Fund, Australia, through the NextGen program, which supports emerging researchers in international agricultural research and development. Naseema holds a Bachelor of Veterinary Science and Animal Husbandry and a master’s degree in Veterinary Pharmacology and Toxicology. Before commencing her PhD, she worked as a veterinary surgeon with the Government of Kerala, India. Her field-based veterinary experience continues to shape her research perspective, with a strong focus on translating fundamental science into practical livestock, animal health, and on-farm outcomes.

Associate Professor Surinder S. Chauhan is the former Director, International, School of Agriculture, Food and Ecosystem Sciences, and Deputy Director, Melbourne India Postgraduate Academy, Faculty of Science, The University of Melbourne. Dr Chauhan’s research program strives to deliver solutions to promote sustainable and efficient livestock production systems. Dr Chauhan has multinational experience in teaching and research in Veterinary and Animal Sciences, and has delivered his services successfully in Australia, India, and the USA. Dr Chauhan is leading Australia–India collaboration for sustainable livestock production between the University of Melbourne, Australia, and ICAR institutes. Dr Chauhan has been invited to speak at various national and international conferences in different countries (Australia, China, Denmark, France, India, Italy, and USA) and has published more than 120 peer-reviewed research papers. Dr Chauhan is committed to endeavour high quality teaching to enrich student learning experiences and supervised 10 PhD and more than 20 Master students.
